# Beyond Macrophages and T Cells: B Cells and Immunoglobulins Determine the Fate of the Atherosclerotic Plaque

**DOI:** 10.3390/ijms21114082

**Published:** 2020-06-08

**Authors:** Harald Mangge, Florian Prüller, Wolfgang Schnedl, Wilfried Renner, Gunter Almer

**Affiliations:** 1 Clinical Institute for Medical and Chemical Laboratory Diagnostics, Medical University of Graz, 8036 Graz, Austria; florian.prueller@medunigraz.at (F.P.); wilfried.renner@medunigraz.at (W.R.); gunter.almer@medunigraz.at (G.A.); 2 Department of Internal Medicine, Practice for General Internal Medicine, 8600 Bruck/Mur, Austria; w.schnedl@dr-schnedl.at

**Keywords:** atherosclerosis, inflammation, B cells, animal model based data, human data

## Abstract

Atherosclerosis (AS) leading to myocardial infarction and stroke remains worldwide the main cause for mortality. Vulnerable atherosclerotic plaques are responsible for these life-threatening clinical endpoints. Atherosclerosis is a chronic, complex, inflammatory disease with interactions between metabolic dysfunction, dyslipidemia, disturbed microbiome, infectious triggers, vascular, and immune cells. Undoubtedly, the immune response is a most important piece of the pathological puzzle in AS. Although macrophages and T cells have been the focus of research in recent years, B cells producing antibodies and regulating T and natural killer (NKT) cell activation are more important than formerly thought. New results show that the B cells exert a prominent role with atherogenic and protective facets mediated by distinct B cell subsets and different immunoglobulin effects. These new insights come, amongst others, from observations of the effects of innovative B cell targeted therapies in autoimmune diseases like systemic lupus erythematosus (SLE) and rheumatoid arthritis (RA). These diseases associate with AS, and the beneficial side effects of B cell subset depleting (modifying) therapies on atherosclerotic concomitant disease, have been observed. Moreover, the CANTOS study (NCT01327846) showed impressive results of immune-mediated inflammation as a new promising target of action for the fight against atherosclerotic endpoints. This review will reflect the putative role of B cells in AS in an attempt to connect observations from animal models with the small spectrum of the thus far available human data. We will also discuss the clinical therapeutic potency of B cell modulations on the process of AS.

## 1. Introduction

Atherosclerosis (AS) is a complex inflammatory disease of the large and medium sized blood vessels with multiple genetic and environmental risk factors. Atherosclerosis remains the leading cause of death worldwide. Circulating low density lipoproteins (LDL) initiate the atherosclerotic process after adhesion to endothelial cells in the intimal space of the vessels. Subsequently, LDL becomes immunogenic through enzymatic and non-enzymatic modifications. ApoB100 peptides activate CD4^+^ T helper cells of the Th1 subtype. An inflammatory response comprises interactions between vascular cells (endothelial, smooth muscle), fibroblasts, immune cells (lymphocytes, antigen presenting monocytes/macrophages), and myeloid cells [1]. This causes a chronic process with formation of vascular lesions—so called atherosclerotic plaques. The plaque can become instable and cause disruption if no effective counter regulatory mechanisms break a series of fatal steps. Disruption is usually associated with hypercoagulation and thrombosis and causes an acute ischemic cardiac and/or cerebrovascular event. So far, innate and adaptive immune cells have been investigated in detail in AS. The fact that human atherosclerotic plaques contain macrophages, dendritic cells, mast cells, and T and B cells has stimulated manifold immunological research activities in AS. So far, it is believed that a reaction to an intimal accumulation of low density lipoproteins represents an essential initial step in the pathologic cascade of AS. In reaction to the lipid accumulation, resident and monocyte-derived macrophages promote lesion development through foam cell transition, which accelerates the inflammatory process. In response to pathogenic antigens, to a great part originating from oxidized lipoproteins, dendritic cells and T cells become activated both locally and systemically. This process induces chronic immune activation, which determines the future fate of the plaque. Stable calcification, inflammatory perpetuation, and decongestion of the structure with bleeding or fibrotic transformation work together and end in different clinical results, i.e., stable steady state with many plaques and hypertension but no dramatic event, or a few aggressive, or even one plaque with dramatic event(s). Notably, so called culprit plaques, which are responsible for clinical end points, are not mandatory for the narrowing of the vessel lumen. These non-obstructive plaques expand rather into the adventitial tissue. From here, very active neovascularization promotes the inflammatory atherosclerotic process by intense cell traffic [2,3,4,5,6]. Being non-obstructive, these plaques remain for a long time clinically asymptomatic, which prevents early diagnosis, a fact that makes them extremely dangerous. Especially, cigarette smoke represents an independent risk factor for plaque development, since chemical constituents of smoke have high oxidant and inflammatory power that can directly induce endothelial damage and potentiate an inflammatory response [7]. Apart from the secured negative influence of smoking, so far unclear systemic triggers are involved in the generation of clinical end points. Infections and other immune response modifying events like nutritional factors [7,8,9] or mitochondrial DNA mutations [10] are under investigation for this. Concerning endothelial activation, Nck1 (non-catalytic region of tyrosine kinase adaptor protein) has been identified recently as playing a role in atheroprone hemodynamics [11].

## 2. B Cells—The Underestimated Players

**Figure 1:**
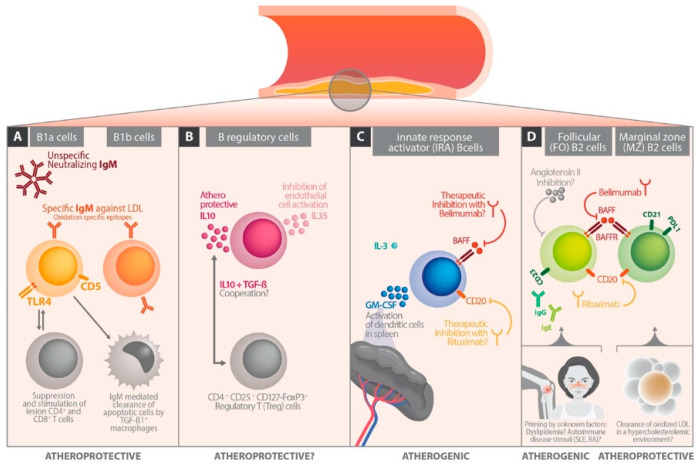
Different B cell subsets found in atherosclerotic lesions of murine models. (**A**) B1a and B1b cells act atheroprotective by production of IgM antibodies against oxidation-specific epitopes. Toll like receptor 4 (TLR4) expressing B1a cells suppress CD4 and CD8 T cells in AS lesions, and increase the TGF-β1 expression on lesion macrophages. TGF-ß1 positive macrophages participate in IgM mediated clearance of apoptotic cells. (**B**) Regulatory B cells act also atheroprotective by secreting IL-10 that influences T regulatory cells. T cell growth factor ß may be involved in this process. IL-35 secretion inhibits mitochondrial reactive oxygen species mediated activation of endothelial cells. (**C**) Innate response activator B cells act atherogenic by producing high amounts of granulocyte–macrophage colony-stimulating factor that activates dendritic cells in spleen. (**D**) The B2 cells form in the spleen and lymph nodes the the follicular (FO) and marginal zone (MZ) B2 cell population. The (FO)B2 cells may act proatherogenic depending on the state of inflammation and the local inflammatory microenvironment (e.g., autoimmune diseases, dyslipidemia). Angiotensin II may influence (inhibit) the activity of (FO)B2 cells. The (MZ)B2 cells are involved in the cholesterol metabolism and act atheroprotective by uptake of oxidized LDL in a hypercholesterolemic environment.

B cells mediate innate, adaptive, humoral, and cellular immune responses. As a unique feature, B cells have hypersomatic mutations and develop occasionally to antibody-producing cells. Antibodies are glycoproteins and form the immunoglobulin classes. Sticking to the surface of the B cell membrane, they serve as the B cell receptor for antigens. When secreted into the extracellular space or circulation, immunoglobulins bind to auto- or foreign antigens. Distinguished by their different C terminus region of the heavy chain (Fc), five main immunoglobulin classes (M, D, A, E, and G) exist. IgG is further divided into four subclasses (IgG_1–4_). Ongoing results showed that IgM antibodies can act as atheroprotectives (Figure 1A), while IgE and IgG antibodies may have proatherogenic features with contradictory data for IgG [12,13]. Concerning IgG subclasses, no robust data of their putative role in chronic inflammation of cardiovascular disease are available. Recent findings show that T cells are not the strict conductors of B cells as formerly thought. Rather data have clarified that the B cells conduct important features of the cellular immune activation in AS [13,14]. The basis for this astonishing perspective is given by the fact that B cells can regulate directly T cell activities via antigen presentation, co-stimulation, and cytokine production [13,14]. In the following, we discuss aspects of these interesting new findings and rank data from animal models as possibly useful in the future as a basis for new diagnostic/therapeutic concepts in the management of human cardiovascular diseases.

While B1a and B1b cells produce protective IgM directed against oxidation-associated LDL epitopes, B regulatory cells attenuate immune responses by production of IL-10. Innate response activator B cells act predominantly atherogenic amongst other effects by activating antigen presentation of dendritic cells in the spleen. Thus, they represent candidates for future immune modulating therapy concepts in AS. B2 cells have inconsistent effects on the atherosclerotic process, whereby long lasting priming factors like dyslipidemia or autoimmune diseases may influence their behaviors.

## 3. B Cell Subsets

B cells are more differentiated than formerly thought, as they were considered to be a kind of servant for the conducting T cell, ready to develop into immunoglobulin producing “factories” if commanded by “clever” T cells. Latterly, with the upcoming perception of their importance, a specific spectrum of functional B cell subsets has been defined. 

**Figure 2:**
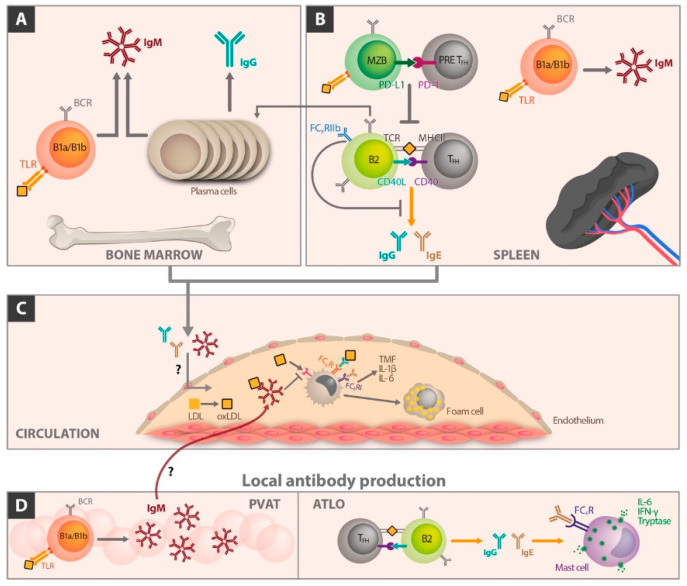
B cell subsets and peripheral immunoglobulin production in atherosclerosis. (**A**) Bone marrow. B1a and B1b cells contact via toll like receptors oxididized LDL and contribute significantly to plasma IgM levels. Plasma cells produce IgG antibodies. (**B**) Spleen. Follicular B2 cells present antigens to follicular B helper T (T_FH_) cells via MHC II/T cell receptor (TCR) interaction and provide costimulatory signaling through CD40-CD40L binding. This process can cause germinal center reactions in which B cells undergo specific maturation and isotype switching to generate high-affinity IgG or IgE antibodies. Additionally, Fcγ receptor IIb (FcγIIb) has been shown to inhibit germinal center derived IgG production in B2 cells. In response to hypercholesterolemia, marginal zone B cells upregulate programmed cell death ligand (PD-L1), which interacts with PD-1 on T_FH_ cells to suppress T_FH_ differentiation. This process attenuates proinflammatory T_FH_/B2 cell interactions. (**C**) During atherosclerosis, LDL accumulation and oxidative transition into oxidized LDL acts proinflammatory. This process attracts monocytes and other immune cells into the sub intimal space. IgM binds oxidized LDL and thus detracts the binding of oxidized LDL to scavenger receptors (SC) on monocytes and macrophages in the lesion. Hence, proinflammatory cytokine secretion and foam cell formation decreases. IgG binding to Fcγ receptors (FcγR) and IgE binding to Fcε receptors (FcεRI) on macrophages can also cause proinflammatory cytokine production. (**D**) IgM, IgG, and IgE antibodies produced peripherally or locally in the perivascular adipose tissue (PVAT) and adventitial tertiary lymphoid organs (ATLOs) enter the lesion and mediate immunomodulatory effects. T_FH_ and B2 interactions in ATLO can result in IgG and IgE antibodies that are proinflammatory. IgE binds to FcεRI of mast cells, resulting in the release of proinflammatory cytokines including IL-6 and interferon-γ.

The B1 cells locate in highest concentrations in the peritoneal and pleural cavities and in lower concentrations in the spleen, bone marrow, and mucosal and adipose tissues. In mice they are divided into the B1a and B1b subset by expression of the surface molecule CD5 (B1a: CD5 positive; B1b: CD5 negative) (Figure 1A). In humans, a subdivision into B1a/b cells has not been found to exist so far. B1a cells are part of the innate immune response and act atheroprotectively by secretion of high amounts of IgM antibodies (Figure 1A). Moreover, the toll like receptor 4 (TLR4)–MyD88 axis may be involved in atheroprotective activities of B1a cells [15]. Hence, TLR4 expressing B1a cells lead to increased TGF-β1 expression on lesion macrophages, reduced apoptotic cells, necrotic cores, and decreased CD4 and CD8 T cell infiltrates in AS plaques. TLR4 related MyD88 is a central player in innate immunity and acts as a canonical adaptor for inflammatory signaling pathways downstream of the TLRs [16]. TGF-ß1 positive macrophages participate in the clearance of apoptotic cells, a process considered as a stabilizing factor in the atherosclerotic lesion. B1a cells also decrease the synthesis of lesion inflammatory cytokines TNFα, IL-1β, and IL-18 [15]. Both B1a and B1b cells can produce specific IgM antibodies against oxidation-specific epitopes on LDL molecules [17] (Figure 1A). Recently, it has been shown in ApoE–/– mice and in a human cohort with active cardiovascular disease that the chemokine CXCR4 (C–X–C motif chemokine receptor 4) plays an important regulatory role in the production of specific IgM antibodies against oxidation specific epitopes on LDL molecules [18]. In this context, it must be noted that B1 cells localized in the bone marrow are involved in IgM production (Figure 2A). The B1 mediated bone marrow IgM synthesis and plasma IgM levels decreased significantly in ApoE–/– mice with B cell-specific knockout of CXCR4 [19]. On the other hand, overexpression of CXCR4 on B1 cells increased plasma IgM levels against oxidation specific epitopes (malondialdehyde-modified LDL) [20]. According to these data from ApoE–/– mice, in a 50-subject human cohort, CXCR4 expression on circulating B1 cells positively correlated with plasma levels of IgM antibodies specific for malondialdehyde-modified LDL and inversely correlated with human coronary artery plaque burden and necrosis [21]. These data identify CXCR4 expression as a critical regulatory factor for the production of IgM against oxidation specific epitopes. That CXCR4 expression on B1 cells was greater in humans with low coronary artery plaque burden suggests a potential targeted approach for immune modulation to limit AS [18,19].

The B2 cells locate in the spleen and lymph nodes where they form follicular and the marginal zone B cell population (Figure 1D). B cell activating factor (BAFF) receptor dependent cells share phenotypic attributes of B1 and B2 cells. Innate response activator (IRA) B cells produce high amounts of granulocyte–macrophage colony-stimulating factors (GM-CSF) *(*Figure 1C). Regulatory B cells (Breg) secrete IL-10 to damp the immune responses [20] (Figure 1B). Additionally produced, IL-35 may inhibit mitochondrial reactive oxygen species mediated activation of endothelial cells [21] (Figure 1B). However, these results come from animal studies, and so far only a few studies investigated the association of B cell subsets with different stages of human AS. Of course, it is difficult to translate B cell interactions from murine AS models to the human system. Some clues for human relevance of murine data come from a genome wide association experiment performed with participants of the Framingham Study. Herein, healthy controls had more B cell genes activated than coronary heart disease patients. This observation suggests a cardiovascular protective effect of B cell activity also in the human system [22]. Further, “pro-atherogenic” CD19^+^CD86^+^ B cells correlated positively, and “atheroprotective” CD19^+^CD40^+^ cells negatively with the risk of developing stroke [23]. Thus, evidence grows that B cells also play an important role in human AS. 

## 4. B Cells in Experimental Atherosclerosis Models

Eighteen years ago, Caligiuri et al. [24] detected that atherosclerotic mice (i.e., hypercholesterolemic ApoE knockouts) showed, after splenectomy, an exacerbation of AS associated with a deficit of splenic B cells. In non-splenectomized mice, antibody titers to oxLDL were increased, and fewer CD4^+^ T cells were found in AS lesions of these protected mice, suggesting a role for T and B cell cooperation [24]. Major et al. [25] observed something similar in a low density lipoprotein (LDL) receptor-deficient (LDL-R(–/–)) mouse model. B cell-deficient LDL-R(–/–) mice on a western diet had strongly decreased total serum antibody and anti-oxidized LDL antibody concentrations which were associated with a 30–40% size increase of AS lesion areas [25]. A subset analysis showed that B2 cells exert more adverse and B1 cells more atheroprotective effects [2]. Ongoing studies refined this observation by finding out that within the B2 population, the marginal zone B cells were the protective ones, whereas follicular B cells acted proatherogenically (Figure 1D). Moreover, innate response activator (IRA) B cells were connected with atherogenic effects by activating classical splenic dendritic cells [26]. The role of B regulatory cells (Bregs) was also discussed [2]. They may have an atheroprotective function via influencing T regulatory cells by IL-10 and T cell growth factor ß production, but this remains to be proven in future studies (Figure 1B).

## 5. The Different Role of Immunoglobulin Classes in Atherosclerosis

Antibody production is a crucial factor of B cell effects in AS. Whereas IgM is considered to act atheroprotectively, the influence of IgG is not clearly understood. IgG antibodies against oxidized LDL (oxLDL) or malondialdehyde-LDL showed positive, negative, or no correlation with AS. These contradictory results, especially for IgG, require new studies to gain a better insight in the complex effects of immunoglobulins. An improved understanding may provide clues for new therapeutic possibilities for myocardial infarction and/or stroke. Emphasizing the murine situation [13], Figure 2 shows aspects of immunoglobulin production relevant for atherosclerotic disease related to different body sites. 

B1 cells of the bone marrow produce significant amounts of IgM directed against oxidation specific epitopes of LDL particles. Toll like receptors are involved in this process and were found positively expressed on the surface of bone marrow B1a and B1b cells. Plasma cells produce IgG antibodies (Figure 2A). 

In the spleen, follicular B2 cells present antigens to T cell receptors (TCR) of follicular B helper T (T_FH_) cells via MHC II and provide costimulatory signaling through CD40–CD40L interaction. This can cause germinal center reactions in which B cells undergo specific maturation and isotype switching to generate high-affinity IgG or IgE antibodies (Figure 2B). Additionally, FcγRIIb has been shown to inhibit germinal center derived IgG production in B2 cells. In response to hypercholesterolemia, marginal zone B cells upregulate programmed cell death ligand (PD-L1), which interacts with PD-1 on T_FH_ cells to suppress T_FH_ differentiation. This process attenuates proinflammatory T_FH_/B2 cell interactions (Figure 2B). During atherosclerosis, LDL accumulation and generation of oxidized LDL act in a proinflammatory manner. 

This process attracts monocytes and other immune cells into the subintimal space (Figure 2C). IgM, IgG, and IgE antibodies produced peripherally or locally in the perivascular adipose tissue (PVAT) and adventitial tertiary lymphoid organs (ATLOs) enter the lesion and mediate immunomodulatory effects. 

So far, the way of access of these immunoglobulins to the AS plaque remains to be clarified. It may occur from the adventitial site, from circulation, or both. IgM binds oxidized LDL and thus detracts binding of oxidized LDL from scavenger receptors on monocytes and macrophages in the lesion. This process reduces proinflammatory cytokine secretion and foam cell formation. IgG binding to Fcγ receptors (FcγRs) and IgE binding to Fcε receptors I (FcεRI) on macrophages can also cause proinflammatory cytokine production (Figure 2C, ATLO). T_FH_ and B2 cell interactions in ATLO can result in IgG and IgE antibodies that are proinflammatory. IgE can bind to FcεRI present on mast cells, resulting in release of proinflammatory cytokines, including IL-6 and interferon-γ. This process stimulates the inflammatory process in the plaque and may be involved in destabilization associated with clinical end points. 

## 6. The Influence of B Cell Differentiation on Atherosclerotic Perpetuation

### 6.1. Follicular B Cells

Circulating B cells originate in large part from splenic follicular B cells. Caligiuri et al. [24] showed that the adoptive transfer of splenic B cells into splenectomized AS-prone ApoE(–/–) mice reversed the splenectomy-induced acceleration of AS. AS mice lacking IgM production (sIgM–/–) developed worse AS paralleled by high IgE antibody levels. These mice had only a few follicular B cells, expressing the low affinity receptor for IgE (CD23). Thus, the clearance of IgE antibodies was low [27]. These observations suggest that follicular B cells may act protectively in AS [12,28]. On the other hand, adoptive transfer of splenic B2 cells into lymphocyte-deficient Rag2–/–γ-chain–/–ApoE(–/–) recipient mice enhanced AS [29] suggesting also a proatherogenic role for follicular B cells. Accordingly, treatment of hypercholesterolemic ApoE(–/–) and LDL-R(–/–) mice with anti-CD20 antibody, which preferentially depletes B2 cells, ameliorated AS [29,30]. In addition, B cell activating factor receptor deficiency in ApoE(–/–) and LDL-R(–/–) mice, or B2 cell depletion using anti-BAFR blocking antibody, reduced AS [31,32]. These observations, in contrast to the former discussed aspects, support an atherogenic role for follicular B cells (see also Figure 1D). The discrepancy may be caused by the fact that the atheroprotective effect of B2 cell depletion works via suppression of proatherogenic T cell responses [30,32]. If T cells are absent, follicular B cells may promote AS [33,34]. Furthermore, the proinflammatory cytokine tumor necrosis factor alpha (TNFα) is an important mediator in the context of B cells. If TNFα is produced by B2 cells, TNFα synthesis by macrophages also increases, which promotes apoptosis and foam cell generation. This process triggers inflammation in the plaque and favors lesion instability [34].

In summary, the role of follicular B cells remains contradictory in AS. There is no simple one-dimensional linkage. Rather, the effects of follicular B cells are complex and in many facets unexplored. Additionally, the fine tuning of the B cell response in the chronic inflammatory milieus is not understood. Furthermore, we do not know how relevant the data generated from murine models (there are no good ones available so far for AS) are for the human system.

Nevertheless, some clues come from the ApoE deficient mouse model of AS that a preexisting priming of the B cell system mediated by a prolonged antigen encountering “experience” may explain some of the so far contradictory observations. Such a stimulation by a dyslipidemic surrounding, e.g., present in ApoE deficient mice, may provoke a “hyper-nervous” response of follicular B cells resulting in proatherogenic effects (Figure 1D). Observations in which transfer of wild-type mice B2 cells had no proatherogenic effects but transfer of B2 cells isolated from ApoE(–/–) donors very well caused proatherogenic effects indicate such a connection [35]. Hence, the inflammatory milieu or the previous “experience” of the B cells may be an important factor in determining the role of follicular B cells in AS. However, once again, these are mouse data that we cannot transfer simply to humans.

Some other important influencing factors are given by subtype (follicular or marginal zone B cells versus Bregs) and numbers of B cells reconstituted in an immune deficient environment and by changes in cytokines and growth factors. For example, B cell deficient mice show very high B cell activating factor levels, which may differentially alter the properties of the transferred B cells. Furthermore, the proatherogenic effect of B cell reconstitution in ApoE(–/–) B cell deficient mice (high B cell activating factor environment) is lost when mice are infused with angiotensin II. This is caused by a switch of the transferred B cells to a regulatory IL-10-producing phenotype dependent on a synergistic effect of BAFF and angiotensin II [36]. Hence, follicular B cells may exhibit different properties in AS depending on the state of inflammation and the local microenvironment (Figure 1D). A possible immune modulating role of angiotensin II remains to be investigated more in detail. Recently, also in human patients, active atherosclerotic vessel disease was brought into connection with decreased frequency of IL-10^+^ B cells [37]. In this study the low frequency of IL-10^+^ B cells correlated directly with a high inflammatory activity and an increased cardiovascular risk. 

The apparent B cell response modulating/suppressing role of angiotensin II may also become of great interest for a better understanding of the pathophysiology of coronavirus infections. As is known, the angiotensin-converting enzyme-2 (ACE2) has been established as the functional host receptor for CoV-NL63, causing the common cold [38], or SARS-CoV and SARS-CoV-2, causing severe acute respiratory syndrome [39]. Many factors have been associated with both altered ACE2 expression and COVID-19 severity and progression, including age, sex, ethnicity, medication, and several co-morbidities, including cardiovascular disease and metabolic syndrome [40]. An insufficient antibody production based on the B cell response modulating/suppressing role of angiotensin II in corona virus infections remains to be elucidated in this context for causing severe outcomes with immune paralysis. 

Current evidence that the above discussed mouse data are also relevant for human cardiovascular disease is coming from new therapies of two autoimmune diseases, namely systemic lupus erythematosus (SLE) and rheumatoid arthritis (RA). Both diseases dispose patients to early AS and increased cardiovascular risk [40,41,42]. These autoimmune diseases provide a truly proinflammatory background that may stimulate a proatherogenic role of follicular B cells. If these diseases are treated with rituximab (i.e., a B cell depleting biological), AS also improves, which is a useful side phenomenon (Figure 1D).

### 6.2. Marginal Zone B Cells

Marginal zone B cells locate in the outer white pulp of the spleen. During maturation they migrate to the marginal sinus. Here they acquire the ability to self-renew and survive for a whole life-span [43]. They move occasionally from the marginal zone to the follicle [44]. Marginal zone B cells participate in early immune responses [45] and can take up oxidized LDL [46] in a hypercholesterolemic environment (Figure 1D). They also express high levels of CD36 [47] and up-regulate CD36 in response to a high cholesterol diet (HCD) [2]. Amongst other functions, CD 36 is involved in cellular fatty acid uptake and utilization [48]. B cell activating factor overexpression leads to an expansion of marginal zone B cells [49] and is associated with decreased plasma cholesterol levels. Possibly, this decreases the size of AS lesions, as shown in high cholesterol diet fed ApoE (–/–) mice [50]. Thus, marginal zone B cells may play a role in cholesterol metabolism (Figure 1D). However, these are again mouse-sourced data, and the relevance for human AS must be made clear in future studies. 

By another mouse model specifically lacking marginal zone B cells, Nus et al. [51] demonstrated that the B2 cell subset can act atheroprotectively (Figure 1D). The protective effects are mediated through the control of T follicular helper cells [51]. Interestingly, this T cell subset was thought to be atherogenic in another study [52]. Responding to a high cholesterol diet, marginal zone B cells migrate into the T cell zone where they bind to pre-T follicular helper cells via programmed death ligand 1 (PDL1) and programmed death receptor 1 (PD1) interaction, impairing their motility and activation, which may mediate an atheroprotective effect (Figure 2B). This observation brings important conceptual changes to our understanding of the immune mechanisms of AS and warns that putative use of rituximab, belimumab, and epratuzumab for the control of AS must be done with caution. These biologicals deplete both follicular and marginal zone B cells, a fact that may include positive but probably also negative therapeutic effects [13].

## 7. Human B Cells

**Figure 3:**
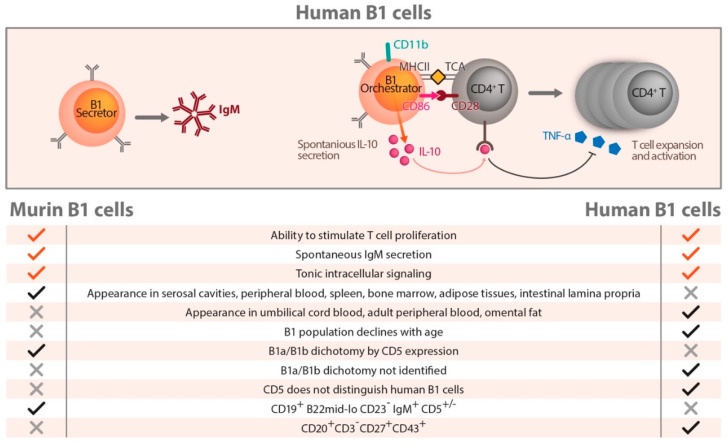
Characteristics of human B1 cells compared to their murine counter parts.

Findings on human B cell subsets lag behind their T cell counterparts because of long missing reagents that clearly identify different cellular subgroups [53]. In contrast to the murine situation, CD 5 was not helpful for the differentiation of B cells in humans (Figure 3). Early investigations in the 1990s used IgD and CD38 expression to classify naive germinal center and memory B cells from tonsils [54]. In the following years, additional surface markers, B cell receptor (BCR) affinity, BCR mutation frequency, and functional assays of immunoglobulin secretion extended the understanding of human B cell populations [53]. These investigations underlined a huge heterogeneity within human B cells not detectable by 1 or 2 surface markers. Moreover, the surface marker expression is a dynamic process rapidly changing over time. A putative human B1 cell subset was identified by Griffin et al. [55] using a reverse engineering approach, in which characteristics of typical murine B1 cell-like ability of T cell stimulation, spontaneous IgM production, and tonic intracellular signaling were used to identify a human B1 equivalent. This subset was defined as CD20^+^CD3^–^CD27^+^CD43^+^ and was found with low frequency in peripheral and umbilical cord blood. Figure 3 shows characteristics of these human B1 cells compared to murine B1 counterparts, including similarities and differences. In contrast to murine B1 cells, no division in B1a and B1b cells exists in humans. Referring to the human situation, as outlined in Figure 3, a division in so called B1 Secretors and B1 Orchestrator cells was introduced by Rothstein et al. [55,56]. B1 Secretors have no or very low CD11b expression and are better able to spontaneously produce IgM (Figure 3, B1 Secretor). B1 cells with high CD11b expression have strong expression of CD86, which enables them to stimulate allogeneic CD4^+^ cells by MHCII–TCR interaction. These B1 Orchestrator cells spontaneously secrete IL-10 (Figure 3, B1 Orchestrator). Interleukin-10 suppresses intracellular TNFα expression of anti-CD3 stimulated T cells in vitro. Referring to this, the B1 Orchestrators very probably modulate T cell activation and proliferation. 

Human B1 cells have been observed in peripheral and cord blood and named B1 Secretors and B1 Orchestrators. Rothstein et al. [56] described them as CD20^+^CD3^–^CD27^+^CD43^+^ cells. These cells produce IgM and stimulate T cells. Further differentiated by the CD11b molecule, human B1 cells with no or low CD11b expression are better able to spontaneously produce IgM (=B1 Secretors). B1 cells with high CD11b expression have higher expression of CD86, a fact that makes them able to stimulate allogeneic CD4^+^ cells and to spontaneously secrete IL-10 (=B1 Orchestrator). Interleukin-10 suppresses intracellular TNFα expression of anti-CD3 stimulated T cells in vitro. Hence, the B1 Orchestrators are potent modulators of T cell activation and proliferation. The table below the graphic in Figure 3 shows homologies and differences between murine and human B1 cell nomenclatures. In contrast to murine B1 cells, no B1a/B1b dichotomy has been described so far in the human B1 cell subset.

Nevertheless, since its discovery, controversy exists concerning this human B1 cell terminology [56,57,58,59,60]. Other studies provided evidence that deeper heterogeneity may exist in the human B1 subset based on chemokine receptor expression [18]. As mentioned before, the expression of CXCR4 on circulating B1 cells is associated with increased amounts of plasma IgM antibodies against anti-oxidation specific epitopes on LDL and is associated negatively with coronary artery plaque burden and stenosis [18]. Hopefully, these data will stimulate research of more in depth characterization of human B1 cell heterogeneity with associated functional attributes. The results may lead to the discovery of new diagnostic biomarkers in human cardiovascular disease. A good basis for this is the fact that human B1 Secretors produce protective IgM, and B1 Orchestrators dampen immune-inflammatory processes [61]. Although still fragmentary, the existing data provide hope that future stronger B cell-focused research in AS may be fruitful to find out new interesting targets to therapeutically control the aggressive components of AS.

## 8. Conclusions

Atherosclerosis is a complex lifetime response to injury that involves both innate and acquired immune response, chronic inflammation, and alterations in the balance of pro- and anti-inflammatory myeloid cells [1]. B cells are clearly more importantly involved than has been supposed so far. Atherosclerosis-associated B cell perturbation may even pave the way for fatal courses of viral infections (e.g., SARS-CoV-2) in elderly people. Additionally, other non-communicable diseases like diabetes mellitus and obesity should be investigated in more detail in this context, because they also render the patient immunocompromised by chronic low grade inflammation. Nutrition and lifelong balanced diets which strengthen the immune response may act as counter regulatory factors with high preventive power [8,9]. Although the understanding of the role of B cells in AS is based to a great part on animal models, it looks also true for the human situation that B lymphocytes have a significant influence on the clinical course of AS and endpoints of cardiovascular disease. Especially, human B1 lymphocytes have complex functions that are the focus of intensive research. Most importantly, they may act protectively in AS by production of neutralizing antibodies of the IgM isotype against oxidation-specific epitopes. The traffic to and localization in vulnerable plaques of B1 cells may give answers to the open question of how immunoglobulins enter and act in vascular AS lesions. Does this occur from the luminal site by crossing the endothelial layer or from adventitia through the vasa vasorum? Do non stenotic vulnerable plaques show a lower number of protective B1 cells? What happens in lymph nodes responsible for the plaque region? Will it make sense to administer protective immunoglobulins therapeutically in AS after early diagnosis of vulnerable lesions well before clinical endpoints? Does this make sense after an event, before an event, or at both time points? Finally, the central question is, to what extent can findings be transferred to a new better therapy of human cardiovascular disease? The latest clinical results (e.g., the CANTOS study) provide hope by showing the impressive potential of immune modulating therapy in cardiovascular disease [62,63]. In summary, the data presented herein indicate the strong potential of B cells as future therapeutic targets for the fight against the most dreadful killers, myocardial infarction and stroke.
